# Transcranial direct current stimulation for post-stroke dysphagia: a meta-analysis

**DOI:** 10.1186/s12984-023-01290-w

**Published:** 2023-12-11

**Authors:** Nerea Gómez-García, Lorena Álvarez-Barrio, Raquel Leirós-Rodríguez, Anxela Soto-Rodríguez, Elena Andrade-Gómez, Pablo Hernández-Lucas

**Affiliations:** 1 Nursing and Physical Therapy Department, University of Leon, Astorga Ave., 24401 Ponferrada, Spain; 2 SALBIS Research Group, Nursing and Physical Therapy Department, University of Leon, Astorga Ave., 24401 Ponferrada, Spain; 3 Pneumology Service, Ourense Hospital, Galician Health Service, 32005 Ourense, Spain; 4 Department of Nursing, University of La Rioja, La Rioja, 26004 Logroño, Spain; 5 Faculty of Physiotherapy, University of Vigo, Campus A Xunqueira, 36005 Pontevedra, Spain

**Keywords:** Deglutition disorders, Pharyngeal diseases, Rehabilitation, Physical therapy modalities, Cerebrovascular disorders, Electric stimulation

## Abstract

**Background:**

Strokes may cause some swallowing difficulty or associated dysphagia in 25–80% of patients. This phenomenon has been linked to increased morbidity and mortality*.* Therefore, the aim of this study was to evaluate the efficacy of transcranial direct current stimulation in patients with dysphagia in post-stroke patients.

**Methods:**

A systematic search in PubMed, Scopus, Web of Science and MEDLINE was conducted. The articles must have to evaluate an intervention that included transcranial direct current stimulation; the sample had to consist exclusively of patients with post-stroke dysphagia; and the experimental design consisted of randomized controlled trial. Difference in mean differences and their 95% confidence interval were calculated as the between-group difference in means divided by the pooled standard deviation. The I^2^ statistic was used to determine the degree of heterogeneity.

**Results:**

Of the 9 investigations analyzed, all applied transcranial direct current stimulation in combination with conventional dysphagia therapy to the experimental group. All the studies analyzed identified improvements in swallowing function and meta-analysis confirmed their strong effect on reducing the risk of penetration and aspiration (Hedges’s g = 0.55). The results showed that participants who received transcranial direct current stimulation significantly improved swallowing function.

**Conclusions:**

Transcranial direct current stimulation has positive effects in the treatment of poststroke dysphagia by improving swallowing function, oral and pharyngeal phase times and the risk of penetration and aspiration. Furthermore, its combination with conventional dysphagia therapy, balloon dilatation with catheter or training of the swallowing muscles ensures improvement of swallowing function.

*PROSPERO registration ID* CRD42022314949

**Supplementary Information:**

The online version contains supplementary material available at 10.1186/s12984-023-01290-w.

## Introduction

Dysphagia is an alteration of the swallowing function consisting of pain and difficulty in passing the food bolus, ingested liquids or saliva from the mouth to the stomach [1]. Eighty percent of cases of dysphagia are of the oropharyngeal type, when it causes alterations at oral, pharyngeal, laryngeal and/or upper esophageal sphincter level [2]. In contrast, esophageal dysphagia involves alterations in the upper esophagus, the body of the esophagus, the lower esophageal sphincter and/or the cardia [3].

The main causes of dysphagia are: diseases of the central nervous system (Parkinson's, multiple sclerosis or stroke) [4], structural alterations (after surgery) or motor disorders (due to weakness or lack of coordination of the musculature) [1, 4]. The incidence of dysphagia after stroke is as high as 78% of cases (depending on the age of the patient and the area of the brain affected) [5]. Many patients recover swallowing spontaneously within the first seven days after stroke [6]. However, up to 50% present with dysphagia at hospital discharge [7] and 11–13% more than six months after stroke [8].

Dysphagia can lead to complications such as malnutrition, dehydration, reduced physical activity, etc. [6] However, the most frequent complication associated with post-stroke dysphagia is aspiration pneumonia (affecting up to 14% of patients) [9]. This, in turn, is associated with a higher risk of mortality [10], longer hospital stays [10] and higher economic costs [11]. Therefore, reducing the degree of swallowing penetration-aspiration in patients with dysphagia reduces serious consequences for their health and even prevents their death [12–14].

Therapeutic options for dysphagia include pharmacological treatment, dietary modifications, compensatory maneuvers, physical therapy methods and conventional dysphagia therapy (CDT) [15]. The latter consists of direct and indirect techniques aimed at strengthening the musculature involved in the swallowing process and ensuring effective and safe swallowing [16]. In addition, different modalities of electrical stimulation for the treatment of post-stroke dysphagia have been studied in the last decade. Pharyngeal electrical stimulation drives neuroplasticity in the pharyngeal motor cortex through direct stimulation of the pharyngeal musculature [17]. Neuromuscular electrical stimulation facilitates muscle contractions during swallowing via electrodes placed on the anterior neck musculature [18]. Finally, repetitive transcranial magnetic stimulation and transcranial direct current stimulation (tDCS) are two non-invasive stimulation options [19, 20]. The former results in depolarization of postsynaptic connections [21]. In contrast, tDCS is a neuromodulator technique that uses direct current to produce changes in neuronal plasticity [22]. Its application can lead to physiological and motor function changes related to the specific stimulation of certain brain regions [23]. In general terms, anode stimulation causes an increase in cortical excitability and cathodal stimulation causes a decrease in cortical excitability [24].

Although several studies have concluded that tDCS has positive effects on swallowing function [19, 20], it is a technique that has not yet been protocolized and for which there is no consensus on the parameters of application and whose ability to reduce the degree of penetration and aspiration has not yet been quantified. For this reason, it was considered necessary to carry out this systematic review and meta-analysis with the aim of evaluating the efficacy of tDCS in patients with post-stroke dysphagia.

## Methods

### Eligibility criteria, information sources and search strategy

This study was prospectively registered on PROSPERO (ID: CRD42022314949) and followed the Preferred Reporting Items for Systematic Reviews and Meta-analyses (PRISMA), the recommendations for their implementation in Exercise, Rehabilitation, Sport Medicine and Sports Science (PERSiST) [25] and the reporting guidelines and the recommendations from the Cochrane Collaboration [26]. The PICO question was then chosen as follows: P—population: patients with post-stroke dysphagia; I—intervention: tDCS; C—control: other rehabilitation techniques and/or CDT; O—outcome: swallowing function, degree of aspiration and/or oral and pharyngeal transit time; S—study designs: randomized controlled trials.

A systematic search of publications was conducted in November 2023 in the following databases: PubMed, Scopus, Web of Science and MEDLINE. The search strategy included different combinations with the following Medical Subject Headings (MeSH) terms: *Deglutition disorders, Stroke, Transcranial direct current stimulation* and *Electric stimulation;* and *Dysphagia* and *Transcranial electric stimulation* as free terms. The search strategy according to the focused PICOS question is presented in Table 1.

**Table 1:** Search strategy according to the focused question (PICO)

Database	Search equation	Results identified	Results selected
PubMed	("Transcranial direct current stimulation" [Mesh]) AND ("Deglutition disorders" [Mesh]) ("Electric stimulation" [Mesh]) AND ("Deglutition disorders” [Mesh]) (“Transcranial electric stimulation") AND ("Deglutition disorders” [Mesh]) (“Transcranial direct current stimulation" [Mesh]) AND ("Stroke" [Mesh]) ("Electric stimulation" [Mesh]) AND ("Stroke" [Mesh]) ("Transcranial electric stimulation") AND ("Stroke" [Mesh]) ("Transcranial direct current stimulation" [Mesh]) AND (“Dysphagia”) ("Electric stimulation" [Mesh]) AND ("Dysphagia") ("Transcranial electric stimulation") AND ("Dysphagia")	1281	5
Medline	(MH “transcranial direct current stimulation”) AND (MH “deglutition disorders”) (MH “electric stimulation”) AND (MH “deglutition disorders”) (”transcranial electric stimulation”) AND (MH “deglutition disorders”) (MH “transcranial direct current stimulation”) AND (MH “stroke”) (MH “electric stimulation”) AND (MH “stroke”) (”transcranial electric stimulation”) AND (“dysphagia”) (MH “transcranial direct current stimulation”) AND (“dysphagia”) (MH “electric stimulation”) AND (“dysphagia”) (”transcranial electric stimulation”) AND (“dysphagia”)	1088	2
Web of Science	TS = (transcranial direct current stimulation) AND TS = (deglutition disorders) TS = (electric stimulation) AND TS = (deglutition disorders) TS = (transcranial electric stimulation) AND TS = (deglutition disorders) TS = (transcranial direct current stimulation) AND TS = (stroke) TS = (electric stimulation) AND TS = (stroke) TS = (transcranial electric stimulation) AND TS = (stroke) TS = (transcranial direct current stimulation) AND TS = (dysphagia) TS = (electric stimulation) AND TS = (dysphagia) TS = (transcranial electric stimulation) AND TS = (dysphagia)	2179	1
Scopus	TITLE-ABS-KEY(transcranial direct current stimulation) AND TITLE-ABS-KEY(deglutition disorders) TITLE-ABS-KEY(electric stimulation) AND TITLE-ABS-KEY(deglutition disorders) TITLE-ABS-KEY(transcranial electric stimulation) AND TITLE-ABS-KEY(deglutition disorders) TITLE-ABS-KEY(transcranial direct current stimulation) AND TITLE-ABS-KEY(stroke) TITLE-ABS-KEY(electric stimulation) AND TITLE-ABS-KEY (stroke) TITLE-ABS-KEY(transcranial electric stimulation) AND TITLE-ABS-KEY (stroke) TITLE-ABS-KEY(transcranial direct current stimulation) AND TITLE-ABS-KEY (dysphagia) TITLE-ABS-KEY(electric stimulation) AND TITLE-ABS-KEY(dysphagia) TITLE-ABS-KEY(transcranial electric stimulation) AND TITLE-ABS-KEY(dysphagia)	5998	2
CINAHL	(MH “transcranial direct current stimulation”) AND (MH “deglutition disorders”) (MH “electric stimulation”) AND (MH “deglutition disorders”) (”transcranial electric stimulation”) AND (MH “deglutition disorders”) (MH “transcranial direct current stimulation”) AND (MH “stroke”) (MH “electric stimulation”) AND (MH “stroke”) (”transcranial electric stimulation”) AND (“dysphagia”) (MH “transcranial direct current stimulation”) AND (“dysphagia”) (MH “electric stimulation”) AND (“dysphagia”) (”transcranial electric stimulation”) AND (“dysphagia”)	879	1

### Study selection

After removing duplicates, two reviewers (X. X.-X. and X.X.-X.) independently screened articles for eligibility. In case of disagreement, A third reviewer (X. X.-X.) finally decided whether the study should be included or not. For the selection of results, the inclusion criteria established that: (a) the interventions applied had to include tDCS; (b) the sample had to consist exclusively of patients with post-stroke dysphagia; and (c) the experimental design consisted of randomized controlled trial. On the other hand, studies were excluded from this review if: (a) they had a non-experimental methodology (reviews, meta-analyses, editorials…); and/or (b) their full text was not available.

After screening the data, extracting, obtaining and screening the titles and abstracts for inclusion criteria, the selected abstracts were obtained in full texts. Titles and abstracts lacking sufficient information regarding inclusion criteria were also obtained as full texts. Full text articles were selected in case of compliance with inclusion criteria by the two reviewers using a data extraction form.

### Data synthesis

Two reviewers mentioned independently extracted data from included studies using a customized data extraction table in Microsoft Excel. In case of disagreement, both reviewers debated until an agreement was reached.

### Data extraction

The data extracted from the included articles for further analysis were: demographic information (title, authors, journal and year), characteristics of the sample (age, sex, inclusion and exclusion criteria, number of participants, and etiology and chronicity of dysphagia), study-specific parameters (study type and objectives), interventions applied (techniques applied, number and frequency of sessions), tDCS application parameters (electrode type and size, electrode position, intensity, stimulation device and application time), follow-up and dropout rates of participants, and results obtained (variables analyzed, instruments used and results throughout the follow-up). Tables were used to describe both the studies’ characteristics and the extracted data.

### Assessment of risk of bias

The Physiotherapy Evidence Database (PEDro) scale and the RoB tool were used to assess the risk of bias [27]. Additionally, the Grades of Recommendations Assessment, Development, and Evaluation (GRADE) approach was employed to assess the quality of the evidence when conducting meta-analysis [28].

### Statistical analysis

Standardized mean differences (SMD) and their 95% confidence interval (CI) were calculated as the between-group difference in means divided by the pooled standard deviation (SD), using the Hedges’ g corrected effect sizes [29]. Hedges’ g was used to allow for the inclusion of smaller studies. While Cohen’s d and Hedges’ g are similar, we used Hedges’ g as it has better performance over Cohen’s d with inclusion of small samples [30]. When these data were not available in the study they were requested via email to the authors. Data were requested in three articles [32–34] for which we did not obtain the information from the corresponding author. Hedges’ g was interpreted using the following cut-off values: 0 to 0.2: very small; from 0.2 to 0.5: small; from 0.5 to 0.8: moderate; and from 0.8: strong [31]. Heterogeneity was measured through I^2^ statistics and explains how much of the variation between studies is due to heterogeneity rather than to chance. Values included between 0 and 40% may suggest “no important” heterogeneity, range 30–60% indicates “moderate” levels, 50–90% may represents “substantial” and 75–100% suggests “considerable” heterogeneity^27^. Analyses were performed with Comprehensive Meta-Analysis (CMA) V2 software (Biostat, NJ).

## Results

### Study selection and characteristics

**Fig. 1:**
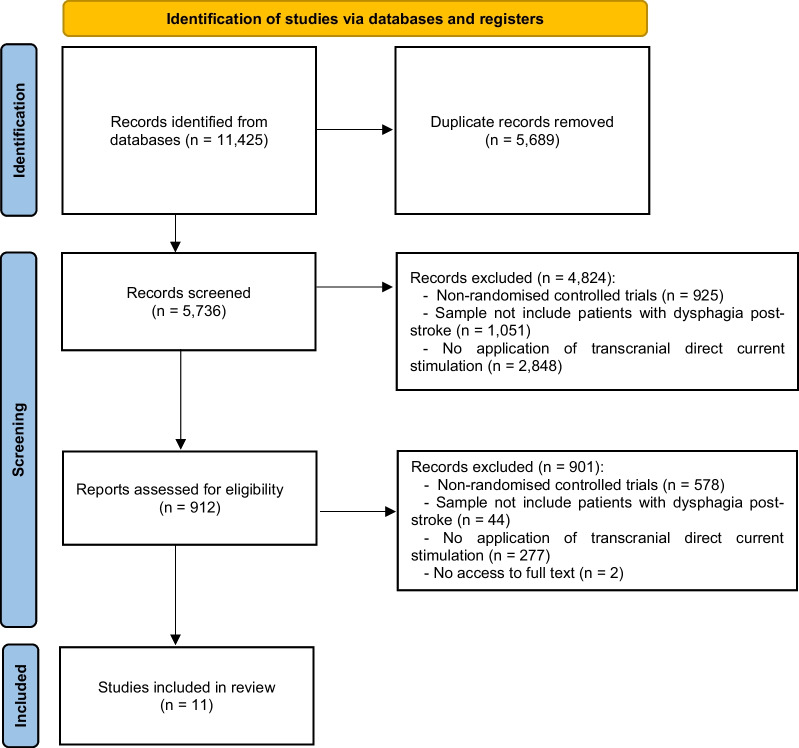
PRISMA flow diagram

A total of 11,425 results were identified. Of these, 5689 were duplicates and 4824 were eliminated after application of the exclusion criteria. Of the 912 remaining results, the full text was analyzed and finally 11 of them were included in this systematic review (Fig. 1).

All the investigations analyzed applied tDCS in combination with CDT to the experimental group [32–42]. The interventions received by the control groups consisted of sham tDCS in combination with CDT [32–35, 37–41] or CDT alone [36, 42].

### Assessment tools

Except one [41], all researchers assessed swallowing function using the *Dysphagia Outcome and Severity Scale* [32–37] and/or videofluoroscopic swallowing analysis [32–37, 39, 40, 42]. In addition, some of them analyzed the *Functional Dysphagia Scale* [36, 39, 40], *Functional Oral Intake Scale* [38, 39, 41], *Penetration Aspiration Scale* [33, 42], *Fiberoptic Endoscopic Dysphagia Severity Scale* [38], *Mann Assessment of Swallowing Ability* [41], *Standardized Swallowing Assessment Scale* [42], *Dysphagia Severity Rating Scale* [37] and the “dysphagia limit” test [38].

### Interventions applied

All investigations evaluated the application of unihemispheric [36, 41, 42] or bihemispheric [32–35, 37–40] anodic tDCS with saline-soaked surface electrodes. Stimulation was performed using one [32–34, 36–42] or two [35] pairs of electrodes.

Ahn et al. [35] used two pairs of electrodes: anodal electrodes were placed bilaterally in the pharyngeal motor cortex and cathodal electrodes in both supraorbital regions in the hemisphere contralateral to the lesion.

The other ten investigations used only two electrodes [32–34, 36–42]. The anode was applied to the uninjured hemisphere [32, 34, 36, 41, 42] (namely the sensory motor cortex [36], supra marginal gyrus [41] or the swallowing sensorimotor cortex [42]), the injured hemisphere [33, 37, 40] (namely the pharyngeal motor cortex [40]) or both [39]. Suntrup-Krueger et al. [38] placed the anode over the center of the cortico-motor swallowing network in the healthy hemisphere in case of cortical stroke patients, whereas the right hemisphere was selected in case of brainstem stroke. The reference electrode (or cathode) was applied on the supraorbital region [32, 34, 37–41] (on the injured side [32, 34, 41] or the opposite side [37–40]), on the shoulder of the injured side [36, 42] or on the uninjured hemisphere (exact location not specified) [33].

In addition, if the patient's condition allowed it, Suntrup-Krueger et al. [38] and Farpour et al. [41] performed conventional swallowing exercises during the application of tDCS. These exercises consisted of different passive and active rehabilitative techniques [41] or swallow maneuvers dry swallows, effort swallows, fluid administration, etc. [38]. Patients who could not perform the exercises waited relaxed and with their eyes open while receiving the stimulation.

The CDT applied included direct methods (dietary modification [35, 40, 41], postural treatment [33, 35, 40, 41], behavioral methods [33, 35, 41], conventional swallowing manoeuvres [32, 40], forced swallows [32], supraglottic swallows [33, 35, 40] and/or effortful swallows [33, 35, 40]) and indirect methods (tactile thermal stimulation [33, 35, 36, 40, 42], external pharyngeal stimulation [36], physical manoeuvres [33], breathing training [42], respiratory muscle training [36], food intake training [42], oropharyngeal stimulation with air-pulse training [42] and with neuromuscular electrical stimulation [42], and/or active exercise of the orofacial muscles [35, 36, 40, 42]). Pingue et al. [33] and Shigematsu et al. [37] specified that patients at risk of aspiration were fed by nasogastric tube and only received indirect therapies. Four of the investigations did not include any description of the techniques or methods included in the CDT [34, 37–39]. Finally, two investigations, in addition to CDT, applied catheter balloon dilatation to all participants [36, 39].

### Results of the research reviewed

The results showed that all participants who received tDCS significantly improved swallowing function [32–42]. This improvement was maintained one [37, 41] and three [40, 42] months after the end of the intervention in all articles where re-evaluations were performed after the end of the intervention.

Control groups receiving sham tDCS and/or CDT also significantly improved their swallowing [32, 33, 35–42], except in one investigation [34]. The improvement in swallowing function was statistically superior in the experimental group in nine investigations [32, 34, 36–42] and similar between the two groups in the remaining two [33, 35].

The risk of aspiration and penetration [33, 34, 42] and oral transit time [34, 40] were significantly reduced with the application of tDCS. Although, Sawan et al. [34] did not identify changes in the control group in either of these two variables. However, Pingue et al. [33], Yang et al. [40] and Wang et al. [42] did observe significant and similar improvements between the two groups of participants.

### Other variables analyzed

Hyoid movement [34], esophageal sphincter spasm [34], activation of the cortico-motor swallowing network [38] and cerebral metabolism [40] were significantly improved with the application of tDCS, but not with CDT alone. In addition, Suntrup-Krueger et al. [38] identified that changes in cortico-motor swallowing network activation were limited only to the stimulated hemisphere.

Cricopharyngeal muscle opening [38] and nutritional (hemoglobin, albumin and prealbumin) and infection (white blood cells and C-reactive protein) [36] indicators were improved in both experimental and control groups, but statistically superior in the former.

The need for nasogastric tube feeding was reduced in 100% of patients who received tDCS (but no participants in the control group could dispense with its use) [37].

Finally, three investigations identified significant relationships between stroke severity (as assessed by the *National Institute of Health Stroke Score*) and duration from stroke onset and the score on the *Dysphagia Outcome and Severity Scale*. Thus, patients with a lower *National Institute of Health Stroke Score* or a more recent stroke had a greater improvement in swallowing function [32, 34, 38].

### Effects on swallowing function

**Fig. 2:**
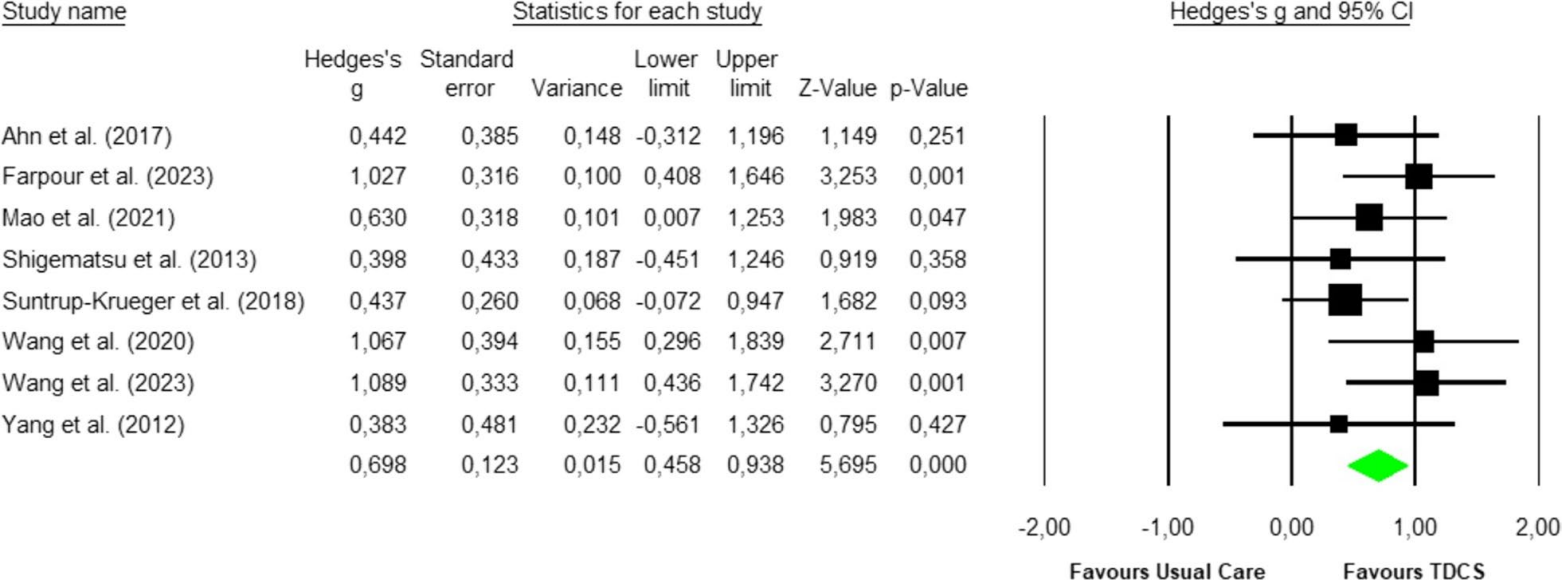
Forest plot for the swallowing function

**Fig. 3:**
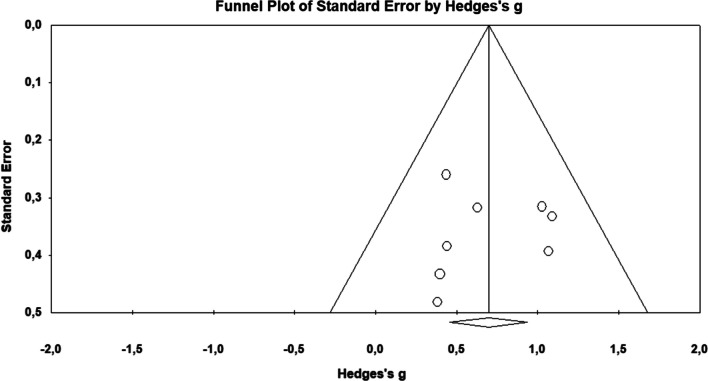
Funnel plot of standard error by Hedges’s g

Eight studies analysed [35–42] were included in the meta-analysis with a total sample size of 273 participants. The Q-test established heterogeneity across the studies and was low (p < 0.58, I^2^ = 0%), and the fixed-effects model was thus used to establish the overall effect size (Fig. 2). Hedges’ g effect size was found to be 0.7, with a variance of 0.02 and 95% CI of 0.46 to 0.94 (p < 0.001). The funnel plot (Fig. 3) showed no evidence of publication bias. Begg and Mazumdar’s test for rank correlation obtained a p-value of 0.45, indicating no evidence of publication bias. Egger’s test for a regression intercept showed a p-value of 0.44, indicating no evidence of publication bias.

### Risk of bias for individual studies

All selected studies scored at least 8 points on the PEDro scale (Table 2), which corresponds to a high level of evidence (Table 3). The risk of bias within individual studies was determined to be critical in three studies (33.3%) [33–35] while seven studies had a low risk of bias (63.6%) [32, 36–39, 41, 42] (Table 4).

**Table 2:** Methodological characteristics of the studies analyzed

Authors	Sample size	Intervention		Intervention duration (number of sessions)	Electrode position	
		Experimental group	Control group		Anode (position in the International System of Electrodes)	Cathode (reference electrode)
Ahn et al. [35] (2017)	26	tDCS + CDT	Sham tDCS + CDT	2 weeks (10)	Bilaterally to the pharyngeal motor cortices, which were 15 cm from central electrode to A1 and 2 cm in the front direction on the right, and from central electrode to A2 in the front direction on the left	Contralateral supraorbital region
Farpour et al. [41] (2023)	44	tDCS + CDT	Sham tDCS + CDT	5 days (5)	Undamaged hemisphere on the supra marginal gyrus (CP5 or CP6 according to 10–10 International Electroencephalogram System)	Contralateral supraorbital region
Kumar et al. [32] (2011)	14	tDCS + CDT	Sham tDCS + CDT	1 week (5)	Undamaged hemisphere (C4-T4 right and C3-T3 left)	Contralateral supraorbital region
Mao et al. [36] (2021)	40	tDCS + CDT	CDT	8 weeks (48)	Sensory motor cortex, uninjured hemisphere (In the middle of C3-T3)	Opposite shoulder
Pingue et al. [33] (2018)	40	tDCS + CDT	Sham tDCS + CDT	10 days (10)	Injured hemisphere (C4-T4 right and C3-T3 left)	Contralateral hemisphere
Sawan et al. [34] (2020)	40	tDCS + CDT	Sham tDCS + CDT	2 weeks (5)	Uninjured hemisphere (C4-T4 right and C3-T3 left)	Contralateral supraorbital region
Shigematsu et al. [37] (2013)	20	tDCS + CDT	Sham tDCS + CDT	10 days (10)	Homolateral hemisphere (15 cm from the central electrode to A1 and 2 cm in the right frontal direction and central electrode to A2 in the left frontal direction)	Contralateral supraorbital region
Suntrup-Krueger et al. [38] (2018)	60	tDCS + CDT	Sham tDCS + CDT	4 days (4)	Centre of the cortico-motor swallowing network (*not described*)	Contralateral supraorbital region
Wang et al. [39] (2020)	28	tDCS + CDT	Sham tDCS + CDT	4 weeks (20)	Bilateral cerebral hemispheres (*not described*)	Contralateral supraorbital region
Wang et al. [42] (2023)	40	tDCS + CDT	CDT	2 weeks (10)	Swallowing sensorimotor cortex of the unaffected side	Contralateral shoulder
Yang et al. [40] (2012)	16	tDCS + CDT	Sham tDCS + CDT	2 weeks (10)	Pharyngeal motor cortex of the lesioned hemisphere (*not described*)	Contralateral supraorbital region

tDCS: Transcranial direct current stimulation. CDT: Conventional dysphagia therapy

**Table 3:**
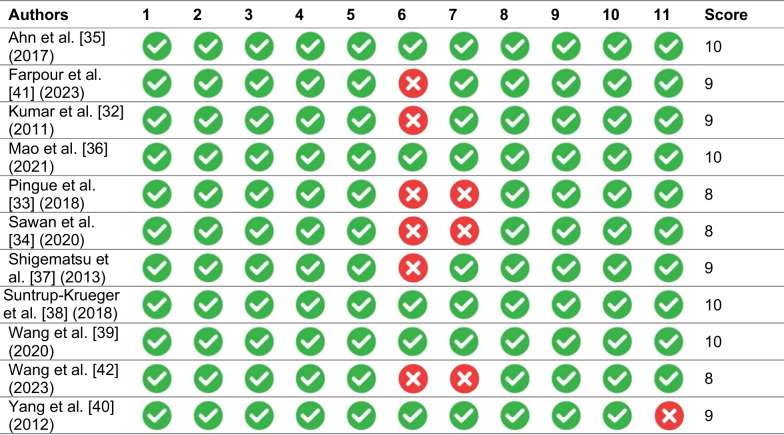
PEDro scale scores

(1) Choice criteria specified (not to be used for scoring); (2) Subjects randomly assigned into groups; (3) Assignment blinded; (4) Groups are similar at baseline with respect to the most important prognostic factors; (5) All subjects were blinded; (6) Therapists were blinded; (7) Evaluators who measured at least one key outcome were blinded; (8) Measures of at least one of the key outcomes were obtained from more than 85% of the subjects initially assigned to the groups; (9) Results were presented for all subjects who received treatment or were assigned to the control group; (10) Results of statistical comparisons between groups were reported for at least one key outcome; (11) Point and variability measures for at least one key outcome

**Table 4:** Risk of bias for included studies (RoB tool results)

Authors	Random sequence (selection bias)^a^	Allocation concealment (selection bias)^b^	Blinding of participants and personnel (performance bias)	Blinding of outcome assessment (detection bias)	Incomplete outcome data (attrition bias)^c^	Selective reporting (reporting bias)^d^	Other bias	Overall
Ahn et al. [35] (2017)	Low	Low	Low	Low	High	Low	Low	High
Farpour et al. [41] (2023)	Low	Low	Low	Low	Low	Low	Low	Low
Kumar et al. [32] (2011)	Low	Low	Low	Low	Low	Low	Low	Low
Mao et al. [36] (2021)	Low	Low	Moderate	Low	Low	Low	Low	Low
Pingue et al. [33] (2018)	Low	Low	Low	High	Low	Low	Low	High
Sawan et al. [34] (2020)	Low	Moderate	Moderate	Low	High	Low	Low	High
Shigematsu et al. [37] (2013)	Low	Low	Moderate	Low	Low	Low	Low	Low
Suntrup-Krueger et al. [38] (2018)	Low	Low	Low	Low	Low	Low	Low	Low
Wang et al. [39] (2020)	Low	Low	Moderate	Low	Low	Low	Low	Low
Wang et al. [42] (2023)	Low	Low	Moderate	Low	Low	Low	Low	Low
Yang et al. [40] (2012)	Low	Moderate	Moderate	Low	Low	Low	Low	Moderate

^a^Risk of bias from confounding was considered critical when confounding was not inherently controlled for (i.e. no or limited adjustment). ^b^Selection bias was critical when selection into the study was very strongly related to intervention and outcome. This occurred when the study included men with diagnoses other than erectile dysfunction. ^c^Risk of bias due to missing data was considered moderate when there appeared to be a substantial amount of missing data. In these cases, the proportions of and reasons for missing data might differ across interventions groups. Of note, the majority of studies did not report on missing data. The risk of bias for these were classified as low, but could also be considered “unknown”. ^d^The studies with a moderate risk for selective outcome reporting were those that did not provided a pre-registered protocol

Additionally, the certainty of the evidence obtained was assessed as moderate for the variable of aspiration and penetration-degree (Table 5).

**Table 5:** Certainty of the evidence (GRADE)

Outcomes	Number of participants (studies)	Risk of bias^a^	Inconsistency^b^	Indirectness	Imprecision	Other considerations	Certainty of the evidence (GRADE)
Aspiration and penetration-degree	273 (8 RCTs)	Moderate	Very low	Low	Low	None	⨁⨁⨁◯ Moderate

RCT: randomized clinical trial; SMD: standardized mean difference

^a^The average risk of bias of the studies according to the RoB tool

^b^Low methodological and statistical heterogeneity among trials (I^2^ < 25%)

⊕: very low; ⊕⊕: low; ⨁⨁⨁◯: moderate; ⊕⊕⊕⊕: high

## Discussion

The aim of this research was to evaluate the efficacy of tDCS in patients with post-stroke dysphagia and the analysis of the results obtained suggests that tDCS improves swallowing function in these patients. Furthermore, the meta-analysis revealed that the effect of tDCS on the degree of penetration and aspiration is moderate.

tDCS improved swallowing function more than CDT in the majority of investigations [32, 34, 36–42]. However, the fact that two studies identified similar effects with both interventions [33, 35] may be due to the fact that they were the only ones that applied tDCS simultaneously in both hemispheres.

Furthermore, the fact that, with the exception of two studies [38, 41], all researchers assessed swallowing function by videofluoroscopic swallowing analysis is a sign of the reliability and validity of the results identified. This test is currently the gold standard for the assessment and management of dysphagia as it objectively measures aspects such as the degree of penetration and aspiration and abnormalities in the swallowing phases [43].

The type of application varied mainly in relation to uni [32, 34, 36–38, 40–42] or bihemispheric [33, 35, 39] stimulation. Of the latter, the two in which both hemispheres were stimulated simultaneously were the only two that did not achieve superior results to isolated CDT [33, 35]. However, Wang et al*.* [39] stimulated first one hemisphere and then the other with results superior to isolated TDC. This could be justified by the fact that most of the pharyngeal musculature involved in the swallowing process is bilaterally innervated and applying CDT bilaterally and alternatively increases cortical excitability in the area [44] (thus leading to beneficial effects in the treatment of dysphagia). Indeed, Li et al*.* [45] demonstrated that tDCS (both unilateral and bilateral) combined with CDT is beneficial for patients with post-stroke dysphagia. However, they concluded that bilateral CDT results in a much greater improvement.

In post-stroke patients it is a therapeutic priority to restore motor function [46]. This could be achieved by increasing the excitability of the injured hemisphere or decreasing that of the uninjured hemisphere [46]. In fact, among the investigations that applied unihemispheric stimulation, the placement of the anode differed depending on whether it was placed in the healthy [32, 34, 36, 41, 42] or injured hemisphere [33, 37, 40]. A previous study by Jefferson et al. [47] demonstrated that tDCS applied to the injured hemisphere improves swallowing function by increasing cortico-bulbar excitability of the pharynx. However, stimulation of the healthy hemisphere has traditionally been chosen for anodal stimulation because it was considered to be the most effective in improving swallowing [48, 49] (especially if the pharyngeal motor cortex was stimulated [15]). In any case, in the present investigation, no differences in the effects on swallowing function of the two therapeutic options were identified. However, it should be noted that the study that managed to improve swallowing with the fewest number of sessions (only four) [38] was the one that defined the placement of the anode according to the location of the stroke.

In parallel, when analyzing the results on oral and pharyngeal transit times, the only intervention that evaluated these variables and failed to improve them was one that stimulated the injured hemisphere [40]. In fact, Suntrup-Krueger et al. [50] in a previous investigation identified that damage in the left hemisphere is associated with oral phase dysfunctions, while in the right hemisphere it causes alterations in the pharyngeal phase. Thus, further studies are needed to investigate the relationship between the site of injury and stimulation and their effect on swallowing transit times.

The investigations that performed re-evaluations confirmed the short- and medium-term benefits of tDCS [33, 38, 41, 42]. This finding is consistent with previous research that has identified persistence of the benefits of tDCS on motor learning and functioning for up to six months after the intervention [51, 52]. tDCS increased brain activity [38] and metabolism [40], especially in the stimulated hemisphere (which was the uninjured hemisphere [38]). In relation to brain activation during swallowing, several studies report that in most patients there is a strong interhemispheric asymmetry [53, 54]. Furthermore, it should also be taken into account that the insula of the right hemisphere and the opercular region are responsible for coordinating oropharyngeal movements and that lesions in these areas are associated with a greater risk of dysphagia and delayed swallowing reflexes [50]. These studies seem to explain why tDCS has long-term benefits, but the functional and microstructural changes need to be further investigated, and electrophysiological studies that explain these improvements are required.

Independently, lesions in the left hemisphere have been identified as having a better response to treatment with electrostimulation [55]. Consequently, it could be that patients with lesions in the left region will have a better response to tDCS, especially those with alterations in the oral phase.

Therefore, this research has identified a number of aspects that favors tDCS to improve dysphagia in post-stroke patients. Firstly, electrode localization can be carried out in a uni (increasing the excitability of the injured hemisphere or decreasing that of the non-injured hemisphere) or bilateral (but never simultaneously) manner. Furthermore, a relationship has been identified between the stimulation site and the improvement in swallowing function, oral phase time and pharyngeal phase time. On the other hand, it has been observed that tDCS has medium to long term effects and that after four sessions significant improvements can be obtained.

Finally, we must recognize that this research has some limitations. The first is the small number of studies included in the meta-analysis. Furthermore, the efficacy of tDCS may be influenced by the different characteristics and application parameters chosen by the researchers (as it is a technique that is not protocolized and there is little consensus on aspects such as the intensity and duration of stimulation). Only the aspiration and penetration degree could be meta-analysed and no other variables related to swallowing function (due to the high variability in assessment methods and the omission of data necessary for the analysis by the original authors). While it is true that in all the studies in the review the intensities used were similar (1–2 mA), specific parameters and times of application should be agreed. This research agrees with previous related meta-analyses on the potential of tDCS to improve swallowing function in these patients [56, 57] but this is the first to meta-analyse the effect of this treatment technique on the severity of penetration and aspiration. However, despite analysing more and more recent studies, the most appropriate application procedure for electrostimulation (left or right hemisphere, injured or uninjured hemisphere, uni- or bihemispheric stimulation, etc.) remains unclear.

## Conclusions

The studies reviewed suggest that tDCS has positive effects in the treatment of poststroke dysphagia by improving swallowing function, oral and pharyngeal phase times and the risk of penetration and aspiration. Efficacy seems to increase when stimulation is applied unilaterally (increasing the excitability of the lesioned hemisphere or reducing the excitability of the non-injured hemisphere) or bilaterally (but not simultaneously).

tDCS is a non-invasive technique that is easy to apply and has very beneficial effects for patients. Furthermore, its combination with CDT ensures improvement of swallowing function.

### Supplementary Information

**Additional file 1:** Table SI. Characteristics and results of the studies analyzed.

## Data Availability

Derived data supporting the findings of this study are available from the corresponding author (X. X.-X.) on request.

## Abbreviations

CDT: Conventional dysphagia therapy
tDCS: Transcranial direct current stimulation
PERSiST: PRISMA implementation in exercise, rehabilitation, sport medicine and sports science
PRISMA: Preferred reporting items for systematic reviews and meta-analyses
