# Supplementary material for: Transcranial direct current stimulation for post-stroke dysphagia: a meta-analysis
Source: J Neuroeng Rehabil. 2023 Dec 11;20:165. doi: 10.1186/s12984-023-01290-w (PMC10712182; doi:10.1186/s12984-023-01290-w)
Supplement: Supplementary file 1 — Additional file 1: Table SI. Characteristics and results of the studies analyzed. [file 12984_2023_1290_MOESM1_ESM.docx]

| **Supplementary table I.** Characteristics and results of the studies analyzed | | | |
| --- | --- | --- | --- |
| **Authors** | **Inclusion criteria** | **Exclusion criteria** | **Results** |
| Ahn et al. (2017) | Age between 18-80 years. Patients with stroke with unilateral cortical or subcortical hemispheric lesion (confirmed by radiodiagnosis). Stroke at least 6 months ago. Diagnosis of dysphagia (DOSS > 5). Inpatients or outpatients receiving CTD 5 times per week. No abnormal response to electrical stimulation. | Active major neurological and/or psychiatric illness (depression, schizophrenia, bipolar or dementia). Depression, schizophrenia, bipolar disorder or dementia. History of stroke. Brain injury in areas other than cortical and subcortical regions. Presence of contraindications for TDCS. Failure to provide signed consent to participate. | Significant improvement in swallowing function (no difference with CTD). |
| Kumar et al. (2011) | *Not described* | Patients with cognitive impairment. History of swallowing disorders. Presence of contraindications for TDCS. | Significant improvement in swallowing function. |
| Lu et al. (2021) | Age between 60 and 80 years. Patients with stroke (confirmed by radiodiagnosis). Diagnosis of dysphagia (with water swallowing test). Patients who met the Chinese sensory stimulation syndromes of wind phlegm and blood stasis. Patients with stable vital signs and gave informed consent. | Dysphagia not caused by stroke. Presence of osteosynthesis. Blood pressure >180 mmHg systolic and/or 12 mmHg diastolic. Diagnosis of cardiac, pulmonary or renal failure. Conscious and/or cognitive impairment. Instability of vital signs. Failure to provide signed consent to participate. | Significant improvement in swallowing function, oral and pharyngeal transit time and risk of aspiration. |
| Mao et al. (2021) | Age between 50 and 80 years. Patients with stroke in the medulla oblongata (confirmed by radiodiagnosis). Patients with water asphyxia, dysphagia and hoarseness. Stroke 2 months ago but less than 12 months. NIHSS score between 2 and 9 points. No cognitive impairment, good compliance. | Diagnosis of other diseases of the nervous system. Presence of dysphagia due to other previous neurological dysfunction and/or radiotherapy to the head and/or neck, critical conditions, cognitive impairment and/or poor behaviour. Failure to provide signe consent to participate. | Significant improvement in swallowing function, oral and pharyngeal transit time, nutritional and infection indicators. |
| Pingue et al. (2018) | Age over 18 years. Patients with unilateral stroke. Stroke during the previous month. Diagnosis of dysphagia between mild and severe (DOSS < 5) NIHSS score < 22 points. | Patients with a history of dysphagia. Presence of other serious clinical conditions, possible contraindications for TDCS, other muscular and/or neurological diseases and/or severe disorder of consciousness. | Significant improvement in swallowing function and risk of aspiration (no difference with CTD). |
| Sawan et al. (2020) | Age between 45 and 60 years. Patients with acute or subacute ischaemic stroke (confirmed by radiodiagnosis). Diagnosis of mild to severe dysphagia. Patients who are vitally stable, oriented and able to follow orders. | History of severe swallowing impairment.  Presence of epilepsy, pregnancy, communication difficulties, neurodegenerative disorder and/or major psychiatric disease, intracranial devices and/or osteosynthesis. Chronic use of medications that affect brain activity. | Significant improvement in swallowing function, oral transit time, aspiration risk and upper hyoid movement.  Significant reduction of oesophageal sphincter spasm. |
| Shigematsu et al. (2013) | Stroke patients. Stroke at least 1 month ago. Diagnosis of chronic or severe dysphagia. Need for nasogastric tube feeding. | Diagnosis of epilepsy, subarachnoid haemorrhage and/or organic neck disease. Presence of severe alteration of consciousness. History of surgery. | Significant improvement in swallowing function. |
| DOSS: Dysphagia Outcome and Severity Scale. CTD: Conventional Therapy for Dysphagia..NIHSS: National Institute of Health Stroke Score. | | | |

| **Supplementary table I.** Characteristics and results of the studies analyzed *(cont.)* | | | |
| --- | --- | --- | --- |
| **Authors** | **Inclusion criteria** | **Exclusion criteria** | **Results** |
| Suntrup-Krueger et al. (2018) | Patients over 18 years of age. Patients with acute ischaemic stroke (confirmed by radiodiagnosis). Stroke less than 24 hours ago. | Pre-existing swallowing difficulties unrelated to stroke. | Significant improvement in swallowing function, risk of aspiration and nutritional status. |
| Wang et al. (2020) | Stroke at least one month ago. Diagnosis of dysphagia due to dysfunction of the cricopharyngeal muscle (confirmed by VFSS). Diagnosis of aspiration by VFSS. Need for nasogastric tube feeding. MMSE score > 23 points. | Presence of severely decreased consciousness, unstable medical conditions, intracranial osteosynthesis. History of epilepsy, dysphagia, radiotherapy for nasopharyngeal carcinoma, head, neck and/or oral cancer and/or other head and neck diseases. | Significant improvement in swallowing function and cricopharyngeal muscle opening. |
| Yang et al. (2012) | Patients with ischaemic stroke. Stroke less than two months ago. Presence of indirect aspiration symptoms and clinical signs of dysphagia. Patient coughing or speaking in a wet voice when asked to drink 5ml of water on swallowing assessment.  Need for nasogastric tube feeding. | History of previous stroke, seizures, severe alcoholism and/or drug abuse. Presence of bilateral brain injury, osteosynthesis, unstable medical conditions, severe language impairment. Diagnosis of depression and/or cognitive deficits. Use of sodium and/or calcium channel blockers and/or N-methyl-D-aspartate antagonists. | Significant improvement in swallowing function and cerebral metabolism.  Significant improvement in oral, pharyngeal and total transit time (no difference with CTD). |
| VFSS: Videofluoroscopic Swallowing Study); MMSE: Mini Mental State Examination. | | | |
